# Antioxidant, α-Amylase and α-Glucosidase Inhibitory Activities and Potential Constituents of *Canarium tramdenum* Bark

**DOI:** 10.3390/molecules24030605

**Published:** 2019-02-09

**Authors:** Nguyen Van Quan, Tran Dang Xuan, Hoang-Dung Tran, Nguyen Thi Dieu Thuy, Le Thu Trang, Can Thu Huong, Yusuf Andriana, Phung Thi Tuyen

**Affiliations:** 1 Division of Development Technology, Graduate School for International Development and Cooperation (IDEC), Hiroshima University, Higashi Hiroshima 739-8529, Japan; nguyenquan26@gmail.com (N.V.Q.); dieuthuykttb@gmail.com (N.T.D.T.); trangle9872@gmail.com (L.T.T.); cth1412@gmail.com (C.T.H.); yusufandriana@yahoo.com (Y.A.); 2 Department of Biotechnology, NTT Institute of Hi-Technology, Nguyen Tat Thanh University, 298A-300A Nguyen Tat Thanh Street, Ward 13, District 4, Ho Chi Minh 72820, Vietnam; 3 Faculty of Forest Resources and Environmental Management, Vietnam National University of Forestry, Xuan Mai, Hanoi 156200 Vietnam; phungtuyen@gmail.com

**Keywords:** *Canarium tramdenum*, bark, antioxidants, α-glucosidase inhibitors, diabetes, phenolics, terpenoids, biological activity

## Abstract

The fruits of *Canarium tramdenum* are commonly used as foods and cooking ingredients in Vietnam, Laos, and the southeast region of China, whilst the leaves are traditionally used for treating diarrhea and rheumatism. This study was conducted to investigate the potential use of this plant bark as antioxidants, and α-amylase and α-glucosidase inhibitors. Five different extracts of *C. tramdenum* bark (TDB) consisting of the extract (TDBS) and factional extracts hexane (TDBH), ethyl acetate (TDBE), butanol (TDBB), and water (TDBW) were evaluated. The TDBS extract contained the highest amount of total phenolic (112.14 mg gallic acid equivalent per g dry weight), while the TDBB extract had the most effective antioxidant capacity compared to other extracts. Its IC_50_ values were 12.33, 47.87, 33.25, and 103.74 µg/mL in 2,2-diphenyl-1-picrylhydrazyl (DPPH), 2,2′-azino-bis (ABTS), reducing power (RP), and nitric oxide (NO) assays, respectively. Meanwhile, the lipid peroxidation inhibition of the four above extracts was proximate to that of butylated hydroxytoluene (BHT) as a standard antioxidant. The result of porcine pancreatic α-amylase inhibition showed that TDB extracts have promising effects which are in line with the commercial diabetic inhibitor acarbose. Interestingly, the inhibitory ability on α-glucosidase of all the extracts was higher than that of acarbose. Among the extracts, the TDBB extract expressed the strongest activity on the enzymatic reaction (IC_50_ = 18.93 µg/mL) followed by the TDBW extract (IC_50_ = 25.27 µg/mL), TDBS (IC_50_ = 28.17 µg/mL), and TDBE extract (IC_50_ = 141.37 µg/mL). The phytochemical constituents of the TDB extract were identified by gas chromatography–mass spectrometry (GC-MS). The principal constituents included nine phenolics, eight terpenoids, two steroids, and five compounds belonging to other chemical classes, which were the first reported in this plant. Among them, the presence of α- and β-amyrins were identified by GC-MS and appeared as the most dominant constituents in TDB extracts (1.52 mg/g). The results of this study revealed that *C. tramdenum* bark possessed rich phenolics and terpenoids, which might confer on reducing risks from diabetes. A high quantity of α- and β-amyrins highlighted the potentials of anti-inflammatory, anti-ulcer, anti-hyperlipidemic, anti-tumor, and hepatoprotective properties of *C. tramdenum* bark.

## 1. Introduction

Diabetes or diabetes mellitus has become a burden for the global economy in recent decades. According to the World Health Organization’s report, this disease and its complications cause substantial economic loss through direct medical costs and loss of work and wages [1]. Among diabetes cases, type 2 diabetes is much more common and chiefly occurs in adults; however, it is being increasingly noted in adolescents [2]. The pathogenesis of type 2 diabetes is currently attributed to endogenous factors such as genetics and metabolic abnormalities and exogenous factors such as behavior and environment [3]. The type 2 diabetes increases blood sugar level which is considered as a typical symptom in diabetic patients. Monitoring and control of hyperglycemia are the most prevalent methods in the treatment of type 2 diabetes nowadays.

As an endogenous toxin, oxidative stress is considered to be an important determinant of type 2 diabetes complications [4]. The causal relation between oxidative stress and type 2 diabetes has been elucidated through molecular mechanisms [5], whereby the overproduction of reactive oxygen species related to hyperglycemia likely leads to an imbalance of the quantity of antioxidants inside the body and eventually, to oxidative stress. On the other hand, the blood sugar level is crucially determined by the act of digestive enzymes such as α-amylase and α-glucosidase. While α-amylase is responsible for breaking down long-chain carbohydrates, α-glucosidase directly converts carbohydrate to glucose in the small intestine. The inhibition of α-glucosidase has been acknowledged as a therapeutic target for the control of postprandial hyperglycemia, as well as type 2 diabetes [6,7]. Therefore, simultaneously providing antioxidants and α-amylase and α-glucosidase inhibitors through nutriments is a potential and feasible method for the management of type 2 diabetes. However, the origin and dose of ingredients should be scrupulously studied before application and production. Additionally, natural products are recommended owing to their long history of medicinal and beneficial effects on human health [8].

Among natural sources, plants have been the most thoroughly scrutinized thanks to their vast diversity and wide distribution across the Earth. It is easy to derive antioxidant and nutrient components from every part of plants as fruits, leaves, stems, and roots which exhibit a wide range of biological effects such as anti-inflammatory, antibacterial, antiviral, anti-aging, and anticancer [9]. Nonetheless, this is also a reason why the potential of plants in treating certain diseases has not yet been fully exploited. *Canarium tramdenanum* Dai & Yakovlev, a synonym of *Canarium pimela* Koenig, a woody tree belonging to Burseraceae family, is not an exception. This plant is widely distributed in subtropical and tropical regions of China and Indochina [10]. In Vietnam, ripe fruits of *C. tramdenum* are commonly used as foods and cooking ingredients, whilst leaves are traditionally used for treating diarrhea and rheumatism [10,11]. In China, *C. tramdenum* or “Chinese black olive” is used in folk medicine as an anti-bacterial, anti-viral, anti-inflammatory, and detoxifying substance [3]. Recently, vasorelaxant and antioxidant activities of the fruits and leaves of this plant have been reported [3,12]. The nutritional compositions of *C. tramdenum* kernels were also documented [13]; however, their biological activities were not investigated. To date, no study on the anti-diabetic property of this plant has been reported. Hence, in this research, we investigated the antioxidant and potential diabetic inhibitory properties of *C. tramdenum* bark extracts through in vitro assays of α-amylase and α-glucosidase suppression. 

## 2. Results

### 2.1. Extraction Yield and Total Phenolic Contents

From 30 g dry TDB, five extracts with different yields were obtained by using an extraction method based on various polarity solvents. The yield of TDBS was 20% (6 g), followed by TDBW (6.67%, 2 g), TDBE (5%, 1.5 g), TDBB (5%, 1.5 g), and TDBH (0.42%, 0.13 g). 

The total phenolic contents (TPC) of TDB extracts were shown in Table 1. It is noteworthy that there was a variation in TPC among extracts which ranged from 20.5 to 112.14 mg GAE/g DW. The total extract TDBS was found to have the highest TPC (112.14 mg GAE/g DW) compared with fractional extracts. The lowest TPC was determined in TDBE extract which accounted for 20.5 mg GAE/g DW. TPC of three fractional extracts TDBE, TDBB, and TDBW (90.62 mg GAE/g DW) were not equal to that of the total extract TDBS. This could be explained by the loss in fractionation and filtration steps (around 21.52 mg GAE/g DW). However, TPC in extracts by higher polar solvents (TDBB and TDBW) were significantly greater than those of extracts by lower polar solvents (TDBE).

### 2.2. Antioxidant Activities 

The antioxidant capacities of TDB extracts by DPPH, ABTS, reducing power and β-carotene bleaching assays are displayed in Table 1.

DPPH is a stable reagent and widely used in most antioxidant tests. The principle of this sensitive assay is based on the reaction of samples with the organic radical DPPH [14]. The antioxidant ability of samples can be observed visually by the discoloration of DPPH (from purple to yellow or colorless) or be determined by the reduced absorbance of the final reaction at 517 nm wavelength. In this study, TDBB extract showed the strongest anti-DPPH radical activity (IC_50_=12.33 µg/mL) which was significantly higher than standard BHT (IC_50_=14.99 µg/mL) and other extracts, see Table 1. 

In the case of the ABTS assay, antioxidants in samples reacting with an organic cation radical named ABTS^•+^ are the basis for this test. In the mixture, with the presence of antioxidants, the nitrogen atom of ABTS^•+^ quenches the hydrogen atom of antioxidants yielding the solution decolorization [15]. The decolorization of ABTS^•+^ solution illustrates the antioxidant capacity of samples. As shown in Table 2, except for TDBE extract, all TDB extracts presented more powerful activity on ABTS assay than the standard BHT. Of which, IC_50_ values of TDBB and TDBW were 1.7-times higher than that of BHT. Additionally, the TDBS extract was 1.3-times the antioxidant activity of BHT.

Reducing power assay or potassium ferricyanide reducing power is based on the competence of antioxidants in converting potassium ferricyanide (Fe^3+^) to potassium ferrocyanide (Fe^2+^). The final reaction with ferric trichloride results in a mixture of Fe^3+^ and Fe^2+^, a blue solution which can be spectrophotometrically determined at 700 nm [14]. An increase in the absorbance (high content of Fe^2+^ in the final reaction) indicates a strong antioxidant activity. By comparing IC_50_ values, see Table 1, the order of the antioxidant capacity of TDB extracts was TDBB (26.24 µg/mL) > TDBS and TDBW (33.25 µg/mL for both) > TDBE (41.60 µg/mL) whilst the IC_50_ value of BHT was 38.34 µg/mL.

In the β-carotene bleaching assay using linoleic acid, the oxidized product (linoleate-free radical and other free radicals) of the linoleic acid peroxidation process can gradually decolorize the β-carotene color by time. The process can be delayed by the presence of antioxidants and the reaction can be recorded at 492 nm. It is apparent that, at the same concentration of 1000 µg/mL, all total extract and fractional extracts of TDB exhibit a similar inhibitory level of lipid peroxidation to the standard BHT, see Table 1. These results demonstrated that antioxidants involved in TDB extracts could negate the free radicals in the system, thereby they could protect β-carotene color from the bleaching process [16].

**Figure 1:**
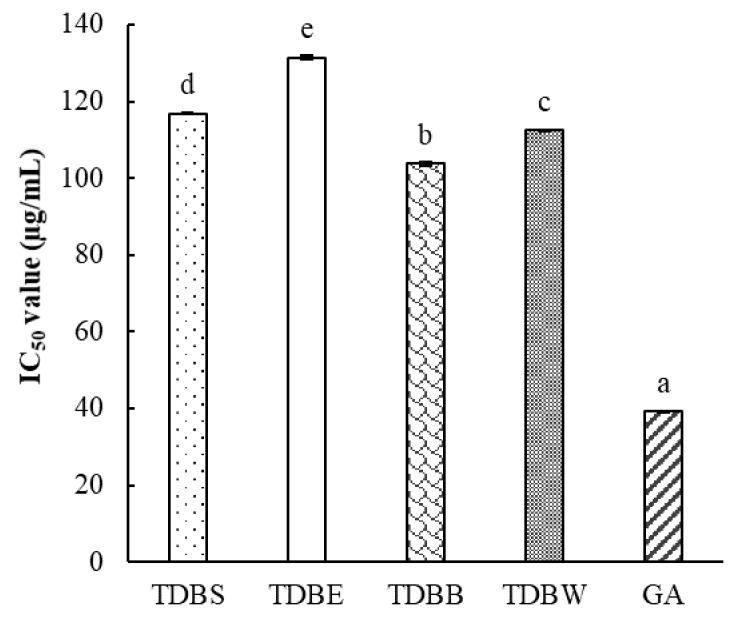
Nitric oxide scavenging activity of TDB extracts. Values are means ± SE (standard error) (*n* = 3); Different letters indicate significant difference at *p* < 0.05; TDBS, *C. tramdenum* total extract; TDBE, ethyl acetate extract; TDBB, butanol extract; TDBW, water extract; BHT: butylated hydroxytoluene; GA: gallic acid.

Sodium nitroprusside in phosphate buffer saline (pH 7.2) generate nitric oxide (NO) which can be spontaneously converted into the more stable forms of nitrate and nitrite ions under the aerobic reaction with oxygen [15,17]. The Griess reagent is used to detect these ions in the mixture by forming the conspicuous pink solution that can be measured at 546 nm. Antioxidants can prevent the formation of nitrate and nitrite ions and, therefore, reduce the absorbance of the reaction. Figure 1 shows that all TDB extracts possess potential NO scavenging activities which are comparable to the standard gallic acid. Among fractional extracts, TDBB expressed the highest antioxidant activity (IC_50_ = 103.74 µg/mL), followed by TDBW (IC_50_ = 112.54 µg/mL), and TDBE (IC_50_ = 131.43 µg/mL). The activity of the total extract TDBS (IC_50_ = 116.80 µg/mL) was significantly higher than that of TDBE but lower than those of TDBB and TDBW. In the body, NO associates with many biological systems including neuronal messenger, vasodilatation, and antimicrobial and antitumor activities [17]. Additionally, the complex interplay between NO production and the pathogenesis of diabetic nephropathy and angiopathy has been interpreted [18,19]. Hence, a nitric oxide scavenging assay is indispensable in the research on the antioxidant and antidiabetic properties of natural products.

### 2.3. α-Amylase and α-Glucosidase Inhibitory Activities

In this study, starch-iodine method was applied to examine the inhibitory effect of TDB extracts on porcine pancreatic α-amylase. The degradation of starch by enzyme activity was visually observed or spectrophotometrically measured at 565 nm based on changing dark-blue color to yellow color of reaction solution. Accordingly, in mixtures with inhibitors, the solution with higher absorption (darker color) signifies the higher inhibitory activity. As shown in Table 2, the anti-α-amylase activity was recorded at all TDB extracts and comparable with the standard acarbose. Among extracts, TDBB extract manifested the highest inhibitory activity (IC_50_ = 257.20 µg/mL) while TDBW represented the lowest one (IC_50_ = 555.02 µg/mL). 

The α-glucosidase inhibitory activity of TDB extracts was assayed using a synthetic substrate pNPG. In physiological buffer (pH 7), α-glucosidase hydrolyzes pNPG to release *p*-nitrophenol, a yellow product that can be measured at wavelength 405 nm [20]. The lower absorbance indicates the stronger suppression on enzymatic activity. All TDB extracts expressed a remarkable inhibition on α-glucosidase activity (Table 2). Of which, the activity of TDBS, TDBB and TDBW extracts were extraordinarily higher than that of standard acarbose. The order of α-glucosidase inhibition is TDBB > TDBW > TDBS > TDBE > acarbose corresponding to IC_50_ values 18.93, 27.27, 28.17, 141.37, and 145.35 µg/mL, respectively.

### 2.4. Correlations between Total Phenolics and Biological Activities

According to Table 3, there was no significant correlation between the total phenolic contents and biological activities of TDB extracts. These results indicated that TPC might not be the main contributor to the antioxidant, anti-α-amylase, and anti-α-glucosidase activities of TDB extracts. On the other hand, except for the β-carotene bleaching assay, all methods showed a strong correlation with each other, regardless of whether they were based on different methods (*p* < 0.05). Especially, antioxidant assays including DPPH, ABTS, reducing power, and nitric oxide were strongly correlated with the α-glucosidase inhibitory assay at *p* < 0.05. Meanwhile, ABTS assay was not significantly associated with the α-amylase inhibitory assay.

### 2.5. GC-MS Results

Phytochemical compositions of TDB extracts analyzed by GC-MS were listed in Table 3. Twenty-four compounds were identified and classified which might be regarded as antioxidants and α-glucosidase inhibitors of TDB extracts.

To the best of our knowledge, except for α-cubebene and (-)-spathulenol that were previously identified in the essential oil of TDB [21], the remaining 22 compounds were the first reported as the principal component of TDB extracts by this study.

Among the identified compounds, phenolics accounted for the highest amount with nine compounds, followed by terpenoids (eight compounds), steroids (two compounds), carbohydrates (glycerol), monosaccharides (levoglucosan), phenylpropanoic acids (ibuprofen methyl ester), carboxylic acids (acetyltributyl citrate) and one unidentified compound. All phenolic compounds were detected in fractional extracts TDBB and TDBW while all terpenoids were identified in the TDBE extract. Although the phenolic components were scant in the total extract TDBS, they were accompanied with greater amounts in the fractional extracts of TDBB and TDBW. This is reasonable when lower polar components have been eliminated through hexane and ethyl acetate solvents. Whereas, 2-propylphenol and acetyltributyl citrate were only detected in fractional extracts TDBB and TDBE, respectively, but were not recognized in the total extract TDBS. Significantly, β-amyrin and α-amyrin were the most dominant compositions in the TDB extract, which showed the highest contents in TDBS (41.45% and 19.17%, respectively) and TDBE (29.51% and 20.18%, respectively) extracts.

### 2.6. Identification of α- and β-Amyrins by GC-MS

The GC-MS analysis on the crystal mixture isolated from F15–F18 of TDBE extract confirmed the presence of α- and β-amyrins (with a 3:4 ratio) as the dominant components. MS data showed that the relative intensity corresponding to the main fragments of α-amyrin were 27, 25, 100, and 13 for peaks at *m/z* 189, 203, 218, and 426, respectively. Meanwhile, β-amyrin data were 15, 52, 100, and 5, respectively at *m/z* values similar to the peaks described above, see Supplementary Materials, Figure S1. The result was coincident with the results of [22,23].

There has been extensive research that validated the diverse pharmacological activity of α- and β-amyrins, including anti-inflammatory, anti-ulcer, anti-hyperlipidemic, anti-tumor, and hepatoprotective actions [24,25]. Also, natural sources of α- and β-amyrins are available which can be easily extracted and isolated in various plants. The most important sources of α- and β-amyrins are Mexican copal (5 mg/g) and *Nelumbo nucifera* (3 mg/g), respectively [25]. Apart from the reported list of plants containing α- and β-amyrins [25], in this study, we supplemented some other sources together with their contents and biological activities, see Table 4. Accordingly, the content of isolated α- and β-amyrins in our study (1.52 mg/g) showed substantial potential in comparison with other plants. Noticeably, *C. tramdenum* bark involves a greater amount of these terpenoids than *C. subulatum* (0.03 mg/g of β-amyrin), a species that belongs to the same *Canarium* genus (Table 4).

## 3. Discussion

*Canarium* is a genus of approximately 100 species in the Burseraceae family [36]. However, very few studies on antioxidant and anti-diabetic potentials were conducted on the species of this genus. Most of them focused on exploiting pharmaceutical and medicinal properties of several species possessing edible fruits. The extracts of *C. album* fruits had potent antioxidants as tannins [37] and phenolics [38] and exhibited effective anti-diabetic properties through antiglycation [39]. The leaf and fruit extracts of *C. odontophyllum* exerted inhibitory effects on diabetic and obese rats [40,41]. The information regarding antioxidant and antidiabetic effects of other inedible parts as stem barks of *Canarium* plants was fragmented and scant. Only stem bark extract of *C. schweinfurthii* was proved to acquire anti-diabetic effects [42]; however, bioactive components of this object were not described. By this study, for the first time, we comprehensively assessed the antioxidant and potential anti-diabetic activities and identified the phytochemicals of *C. tramdenum* bark’s extracts. All extracts presented a similar antioxidant level to BHT, see Table 1, a well-known antioxidant compound and also commonly used in food additives [43]. The TDBB extract was even more active than BHT in DPPH, ABTS, and reducing power assays, see Table 1. Furthermore, TDB extracts showed a potential effect on the restraint of α-amylase activity and exerted much more powerful α-glucosidase inhibition than acarbose—a standard inhibitor that is ordinarily used in the clinical practice of diabetes treatment [44].

The antioxidant property is mainly characterized by phenolic contents [45] which can effortlessly release hydrogen donors to naturalize free radicals. Meanwhile, in α-amylase and α-glucosidase inhibition, both terpenoids and phenolics might play crucial roles [46]. In this study, we found that although the total extract of TDBS showed the highest phenolic content, its biological activities were not stronger than others, see Table 1 and Table 2 and Figure 1. We assumed that phenolics might not be the only contributors toward the antioxidant and antidiabetic properties of TDB extracts. The examination of correlations between phenolics and biological activities of TDB extracts, see Table 3, revealed that the total amount of phenolics might not be the determinant of the biological activities of TDB extracts. The other factors such as functional groups of individual compounds may play more important roles in this case. In particular, the number of free hydrogen donors determines the antioxidant activity, the position of hydroxyl groups, methoxy groups, and lactone rings in the structure of compounds induce inhibitions of α-amylase and α-glucosidase and even the interaction among compounds in an extract may result in differences in biological activities. In this study, we hypothesized that the synergistic interaction between phenolic and terpenoid components might result in the greater biological activities for TDB extracts. However, the composition of phenolics seemed to be more essential when TDBB and TDBW extracts contained a high content of phenolics but not terpenoids and presented stronger antioxidant and anti-α-glucosidase activities than other extracts, see Table 5. Integrating with correlation results, we suggested that the presence of some phenolics, apart from other components, might be the major factor that determines the biological activity of TDB extracts. On the other hand, a previous study demonstrated the potential antihyperglycemic and hypolipidemic effects of α- and β-amyrins [47]. However, the successful isolation of α- and β-amyrins should be further approached to investigate the role of the two compounds in the TDB extracts, see Table 3. Moreover, the high yield of the isolation of these bioactive compounds in this study suggested the potential practical use of TDB extracts, see Table 5. Therefore, although the GC-MS results showed most of the major components of TDB extract, more sensitive methods such as the ultra-high performance liquid chromatography integrated with tandem mass spectrometry (UPLC-MS-MS) should be conducted to affirm and quantify phenolic compounds and other active components. Additionally, the subsequent in vivo tests and clinical trials should be implemented in order to certify the antidiabetic property of this prospective plant. 

## 4. Materials and Methods 

### 4.1. Materials and Instrumentations

Stem bark of *C. tramdenum* (TDB) was collected at 21.07°N, 106.10°E, Bac Ninh province, Vietnam in 2016. The species was identified at a study field based on Vietnam Plant Data Center (http://www.botanyvn.com) and Plants Database Missouri Botanical Garden, United States (TROPICOS—http://www.tropicos.org). The specimen with voucher number TDB-J2016 was deposited at the Plant Physiology and Biochemistry Laboratory, IDEC, Hiroshima University, Japan.

All extraction solvents including methanol, hexane, ethyl acetate, and butanol were purchased from Junsei Chemical Co., Ltd., Tokyo, Japan. The analytical reagents for antioxidant assays were acquired from Kanto Chemical Co., Inc., Tokyo, Japan while those of enzymatic assays were procured from Sigma-Aldrich, St. Louis, MO, USA.

A vacuum evaporator (Rotavapor^®^ R-300, Nihon Buchi K.K., Tokyo, Japan), a microplate reader (Multiskan^TM^ Microplate Spectrophotometer, Thermo Fisher Scientific, Osaka, Japan), and GC-MS system (JMS-T100 GCV, JEOL Ltd., Tokyo, Japan) were the main instruments used in this study.

### 4.2. Sample Preparation and Extraction

After air-drying for 2 days, the outer layer of TDB was scraped to remove all fungi and lichen. Then, the sample was washed with 1% sodium hypochlorite (NaOCl) and water. After blotting with tissues, bark samples were dried at 45 °C for 1 week by an oven, then ground to make the powder. 

The TDB sample (30 g) was extracted using 1 L of methanol in one week. Afterwards, dry methanol crude extract (TDBS) was blended with distilled water (200 mL), then successively fractionated by an equal volume of hexane, ethyl acetate, and butanol. Each fractionation step was conducted three times. After filtrations, they were separately dried under vacuum by an evaporator at 50 °C. Eventually, five dried extracts including TDBS (total extract), hexane (TDBH), ethyl acetate (TDBE), butanol (TDBB), and water (TDBW) extracts were obtained as fractional extracts. They were preserved in sterilized vials and kept in a refrigerator (−20 °C) for further tests on the physical properties, biological activities, and identification of phytochemical components.

### 4.3. Determination of Total Phenolic Content

The total phenolic content (TPC) was calculated according to the Folin-Ciocalteu method described previously [48]. Specifically, 20 µL of extract was homogenized with 100µL of Folin-Ciocalteu’s reagent (10%) and 80µL of 7.5% Na_2_CO_3_ (*w*/*v*), respectively. The mixture was then shaken and incubated at room temperature for 30 min. The absorbance was measured at 765 nm using a microplate reader. TPC was expressed as mg gallic acid equivalent per g dry weight of sample (mg GAE/g DW).

### 4.4. DPPH Radical Scavenging Assay

DPPH assay was evaluated following Elzaawely et al. [49] with a slight modification. Fifty microliters of extract were mixed with 50 µL of DPPH solution (0.2 mg/mL) and 100 µL of 0.1 M acetate buffer (pH 5.5). Thereafter, the combination was incubated for 20 min in darkness at room temperature. The absorbance was recorded at 517 nm using a microplate reader. BHT was used as a standard reference while pure methanol was used as a negative control. The IC_50_ value was calculated as the concentration required to reach a 50% reduction of DPPH.

### 4.5. ABTS Radical Cation Decolorization Assay

2,2′-Azino-bis radical cation (ABTS^•+^) decolorization was measured as described by Pellegrini et al. [50] with minor modifications. ABTS^•+^ solution was prepared by mixing aqueous ABTS (7 mM) solution with 2.45 mM potassium persulfate (1:1 *v*/*v*) and incubating in darkness at room temperature for 16 h. The working solution was then obtained by diluting ABTS^•+^ solution in methanol to an absorbance of 0.70 ± 0.05 at 734 nm. In each well of a 96 well-plate, 25 µL of TDB sample was added to 200 µL of the working solution. After a slight shake, the plate was covered by an aluminum foil and kept at room temperature for 30 min. Subsequently, the absorbance was recorded by a Multiskan^TM^ Microplate Spectrophotometer (Thermo Fisher Scientific, Osaka, Japan). The ABTS radical decolorizing activity was calculated by the following formula

ABTS radical decolorizing activity (%) = (1 − A_sample_/A_control_) × 100

where A_sample_ is the absorbance of reaction with momilactones or positive control (BHT) and A_control_ is the absorbance of reaction without momilactone or positive control. The IC_50_ value was determined as the concentration needed to bleach 50% of ABTS^•+^.

### 4.6. Reducing Power Assay

The reducing capacity was carried out referring to the modified method of Minh et al. [51] with a 10-times reduction in the volume of each reaction component. Initially, 100 µL of sample, 250 µL of 0.2 M phosphate buffer (pH 6.6), and 250 µL of potassium ferricyanide (1%, *w*/*v*) were combined and incubated at 50 °C for 30 min. Subsequently, the reaction mixture was stopped by 500µL of trichloroacetic acid (10%, *v*/*v*) and centrifuged for 10 min at 4000 rpm. Eventually, 100µL of the supernatant was double diluted with distilled water (100 µL) followed by adding 20 µL of ferric chloride solution (0.1%, *w*/*v*). After shaking, the absorbance of the resulting mixture was read at 700 nm by a microplate reader. BHT was used as a standard reference. The IC_50_ value was calculated as mentioned above.

### 4.7. β-Carotene Bleaching Assay

The β-Carotene bleaching activity of TDB extracts was assayed using a β-carotene linoleate bleaching system [52] with trivial adjustments. Briefly, a mixture of 1mL β-carotene (200 µg/mL in chloroform), 20 µL linoleic acid, and 200 mg Tween 40 was evaporated at 40 °C. Afterwards, a volume of 50 mL of oxygenated water was slowly added and the mixture which was then vigorously shaken to form a stable emulsion. The emulsion was freshly prepared before each experiment. In each well of a 96 well-plate, 25 µL of sample or control (1000 µg/mL in methanol) and 200 µL of the emulsion solution were blended. The reaction was incubated at 45 °C and the absorbance was recorded at 492 nm every 15 min up to 180 min. The lipid peroxidation inhibition (LPI) was calculated as:

LPI inhibition (%) = A_180_/A_0_ × 100

where A_0_ is the absorbance of reaction at the zero-minute time and A_180_ is the absorbance of reaction at the 180-min time. Methanol was used as negative control, whilst BHT was the positive control.

### 4.8. Nitric Oxide Scavenging Assay

Nitric oxide scavenging was measured following the method of Govindarajan et al. [53] with some modifications. At the beginning, 100 µL of sample and 100 µL of 5 mM sodium nitroprusside in 0.1 M phosphate buffer saline (pH 7.2) were assimilated and incubated for 30 min at 25 °C. In the next step, 100 µL of the above mixture was blended with 100 µL of Griess reagent (1% sulfanilamide, and 0.1% naphthyl ethylenediamine dihydrochloride in 2% phosphoric acid). The absorbance decrease of the resulting purple solution was recorded at 546 nm by a microplate reader. Pure methanol was used as a negative control while gallic acid was used as a positive reference. The IC_50_ value was obtained in the same way as described above.

### 4.9. Porcine Pancreatic α-Amylase Inhibition Assay

The α-amylase inhibitory activity was assayed based on the starch-iodine method [54]. α-Amylase solution (2 mg/mL) from porcine pancreas (type VI-B, Sigma-Aldrich, St. Louis, MO, USA) and TDB extracts were dissolved in 0.2 M phosphate buffer saline (pH 6.9). Iodine (0.25 mM) and soluble starch (0.5%) solutions were prepared in deionized water. Firstly, 20 µL of α-amylase was mixed and incubated with 20 µL of the test sample at 37 °C in 10 min. After that, 30 µL of starch solution was added and then incubated for 8 min. The reaction was suspended by adding 20 µL of 1 M HCl, followed by 100 µL of iodine solution. The resulting mixture was measured at a wavelength of 565 nm by a microplate reader. The inhibition percentage and IC_50_ value were calculated as described previously [54]. Acarbose was used as a positive reference.

### 4.10. α-Glucosidase Inhibition Assay

The anti-α-glucosidase activity of TDB extracts was measured using a modified version of the method described by Johnson et al. [55]. In brief, 40 µL of TDB extract in 0.1 M potassium phosphate buffer (pH 7) were pre-mixed with 40 µL of 0.5 U/mL α-glucosidase (from *Saccharomyces cerevisiae*, Sigma-Aldrich, St. Louis, MO, USA). After 6 min of incubation at 25 °C, a 20 µL aliquot of 5 mM p-nitrophenyl-α-D-glucopyranoside (pNPG) substrate in the buffer was added and the mixture was incubated for another 8 min. Finally, the reaction was terminated by adding 100 µL of 0.1 M Na_2_CO_3_, and absorbance was recorded at 405 nm. Inhibition percentage was calculated by:

% inhibition = (1 − A_sample_/A_control_) × 100

where A_sample_ is absorbance of the reaction with samples or positive controls (acarbose) and A_control_ is absorbance of reaction with 10% methanol. The enzymatic inhibitory activity of TDB extracts was expressed as IC_50_ value as well.

### 4.11. Identification of Phytochemical Component by GC-MS

The phytochemical components of TDB extracts were identified by GC-MS analysis. The GC-MS system (JMS-T100 GCV, JEOL Ltd., Tokyo, Japan) with an autosampler coupled with a 30 m x 0.25 mm I.D. x 0.25 μm film thickness DB-5MS column (Agilent Technologies, J & W Scientific Products, Folsom, CA, USA). A concentration of 1000 µg/mL of each TDB extract was used for the initial injection. Helium was used as a carrier gas at split ratio 5:1. The GC oven conditions were as follows: The initial temperature was 50 °C without hold time, the boosted temperature was up to 300 °C at 10 °C /min, and held for 20 min. The injection port and detector temperature were set at 300 °C and 320 °C, respectively. The mass range scanned from 29 to 800 amu. The control of the GC-MS system and the confirmation of analytes were conducted using JEOL’s GC-MS Mass Center System Version 2.65a [56].

### 4.12. Isolation of Bioactive Compounds α-Amyrin and β-Amyrin from TDBE Extract

By preliminarily screening the GC-MS results, we found that two major compounds in TDB extracts were tentatively the pentacyclic triterpenes α-amyrin and β-amyrin. Therefore, we implemented an isolation of these two compounds by column chromatography. In brief, The TDBE extract (1.2 g) was premixed with 5 g silica gel (70-230 mesh, Merck, Darmstadt, Germany). The mixture was then loaded onto a normal phase silica gel (40 g) column (2 × 50 cm). The mobile phase was 100% hexane and hexane:ethyl acetate (98:2, *v*/*v*) which yielded 8 fractions (F1–F8) and 12 fractions (F9–F20), respectively. The elution step for every fraction was 100 mL. Fractions F15–F18 afforded a mixture of colorless crystal (45.6 mg) which was subsequently identified as α-amyrin and β-amyrin (75% purity) by GC-MS, see Supplementary Material, Figure S1.

### 4.13. Statistical Analysis

Data were elaborated on the Minitab 16.0 software (Minitab Inc., State College, PA, USA). All assays were thrice implemented, and results were displayed as means ± standard errors (SE). Significant differences among tests were determined by one-way ANOVA using Tukey’s test at *p* < 0.05. Pearson’s correlation coefficients among total phenolics, antioxidant, anti-alpha amylase, and glucosidase activities of TDB extracts (*n* = 3 for each extract) were calculated by using the same software.

## 5. Conclusions

This study documented that *Canarium tramdenum* bark possessed potent antioxidant and α-glucosidase inhibitory activity in in vitro trials. It was found that the fractions of ethyl acetate, butanol, and water extracts enriched with terpenoids and phenolics appeared as a promising source of natural antioxidants, α-amylase, and α-glucosidase inhibitors. In vivo tests and clinical trials should be elaborated to affirm the bioavailability of *C. tramdenum* bark for the development of food additives and supplements to reduce the risks from type 2 diabetes. The isolation of novel constituents, as well as investigations on potent pharmaceutical properties of *C. tramdenum* bark, should also be conducted. The contribution of α- and β-amyrins to biological activities of *C. tramdenum* bark should be further investigated.

## Figures and Tables

**Table 1:** Total phenolic contents and antioxidant activities of TDB extracts.

Samples	TPC (mg GAE /g DW)	DPPH Assay IC_50_ (µg/mL)	ABTS Assay IC_50_ (µg/mL)	RP Assay IC_50_ (µg/mL)	βC Assay LPI (%)
TDBS	112.14 ± 1.19 ^a^	15.41 ± 0.10 ^c^	62.21 ± 1.78 ^b^	33.25 ± 0.04 ^b^	86.12 ± 0.98 ^ab^
TDBE	20.50 ± 0.60 ^c^	22.23 ± 0.09 ^e^	76.96 ± 1.04 ^c^	41.60 ± 0.03 ^d^	87.52 ± 0.73 ^a^
TDBB	36.57 ± 0.36 ^b^	12.33 ± 0.02 ^a^	47.87 ± 0.12 ^a^	26.24 ± 0.02 ^a^	86.75 ± 0.84 ^ab^
TDBW	33.55 ± 0.48 ^b^	16.45 ± 0.07 ^d^	45.25 ± 0.17 ^a^	33.25 ± 0.06 ^b^	84.09 ± 0.56 ^b^
BHT	-	14.99 ± 0.06 ^b^	80.26 ± 1.11 ^c^	38.34 ± 0.01 ^c^	86.67 ± 0.33 ^ab^

Data express means ± SE (standard error); Different superscript letters (^a,b,c,d,e^) in a column indicate significant differences at *p* < 0.05; -: not measured; TPC, total phenolic contents; GAE, gallic acid equivalent; DW, dry weight; DPPH, 2,2-diphenyl-1-picrylhydrazyl; ABTS, 2,2′-azino-bis; RP, reducing power; NO, nitric oxide; βC, β-carotene bleaching; TDBS, *C. tramdenum* total extract; TDBE, ethyl acetate extract; TDBB, butanol extract; TDBW, water extract; BHT: butylated hydroxytoluene.

**Table 2:** α-Amylase and α-glucosidase inhibitory activities of *C. tramdenum* bark (TDB) extracts.

Sample	α-Amylase Inhibition IC_50_ (µg/mL)	α-Glucosidase Inhibition IC_50_ (µg/mL)
TDBS	359.32 ± 6.73 ^c^	28.17 ± 0.12 ^c^
TDBE	491.23 ± 2.49 ^d^	141.37 ± 0.86 ^b^
TDBB	257.20 ± 1.15 ^b^	18.93 ± 0.07 ^e^
TDBW	555.02 ± 9.10 ^e^	25.27 ± 0.12 ^d^
Acarbose	80.26 ± 0.24 ^a^	145.35 ± 0.62 ^a^

Data express means ± SE (standard error); Different superscript letters (^a,b,c,d,e^) in a column indicate significant difference at *p* < 0.05; TDBS, *C. tramdenum* total extract; TDBE, ethyl acetate extract; TDBB, butanol extract; TDBW, water extract.

**Table 3:** Pearson’s correlation coefficients between total phenolics and biological activities.

	TPC	DPPH	ABTS	RP	NO	βC	AG
**DPPH**	0.24	-	-	-	-	-	
**ABTS**	−0.15	0.68*	-	-	-	-	
**RP**	0.10	0.99*	0.72 *	-	-	-	
**NO**	0.08	0.96*	0.86 *	0.97 *	-	-	
**βC**	−0.08	−0.10	−0.52	−0.10	−0.26	-	
**AG**	0.23	0.95*	0.85 *	0.94 *	0.98 *	−0.33	
**AA**	0.20	0.85*	0.25	0.84 *	0.70 *	0.30	0.65 *

* a significance at *p* < 0.05; TPC, total phenolic contents; DPPH, 2,2-diphenyl-1-picrylhydrazyl assay; ABTS, 2,2′-azino-bis assay; RP, reducing power assay; NO, nitric oxide scavenging assay; βC, β-carotene bleaching assay; AG, α-glucosidase inhibitory assay; AA, α-amylase inhibitory assay.

**Table 4:** Natural sources of α-amyrin and β-amyrin and their principal biological activities.

Plant Species	Plant Parts	Quantity	Biological Activities	References
*Protium kleinii*	Resin	Mixture of α-,β-amyrins (1:1) 2.4 mg/g	Antinociception against the visceral pain in mice	[26]
*Protium heptaphyllum*	Trunk wood resin	Mixture of α-,β-Amyrins	Gastroprotective, anti-inflammatory and hepatoprotective properties	[24,27,28]
*Symplocos cochinchinensis*	Leaves	β-Amyrin 0.17 mg/g	Antioxidant and free-radical scavenging effects	[29]
*Alstonia boonei*	Stem barks	β-Amyrin 0.08 mg/g	Anti-inflammatory activity	[30]
*Memecylon umbellatum*	*nm*	*nm*	α-Glucosidase inhibitory activities	[31]
*Melastoma malabathricum*	Leaves	α-Amyrin 0.06 mg/g	*nm*	[32]
*Maesobotrya barteri*	Aerial parts	β-Amyrin	Antimicrobial activity	[33]
*Swertia longifolia*	Aerial parts	α-Amyrin 0.01 mg/g; β-Amyrin 0.02 mg/g	α-Amylase inhibitory activity	[34]
*Canarium subulatum*	Stem bark	β-Amyrin 0.03 mg/g	Anti-herpetic activity	[35]
*Canarium tramdenum*	Barks	Mixture of α-,β-amyrins (3:4) 1.52 mg/g	*nm*	This study

*nm*, not mentioned

**Table 5:** Major phytochemical components of TDB extracts analyzed by GC-MS.

No.	Identified Compounds	RT (min)	Chemical Classification	Peak Area in Extracts (%)			
				TDBS	TDBE	TDBB	TDBW
1	Glycerol	4.62	Carbohydrates	<	-	-	37.73
2	2-Methoxyphenol	6.39	Phenolics	1.19	-	1.57	3.63
3	Pyrocatechol	7.88	Phenolics	1.28	-	19.31	6.66
4	4-Methylcatechol	9.21	Phenolics	<	-	1.67	1.38
5	2,6-Dimethoxyphenol	10.08	Phenolics	<	-	-	1.43
6	2-Propylphenol	10.35	Phenolics	-	-	3.54	-
7	α-Cubebene	10.58	Terpenoids	0.67	<	-	-
8	3,4-Dimethoxyphenol	11.05	Phenolics	<	-	-	5.22
9	Levoglucosan	11.82	Monosaccharides	<	-	4.30	1.92
10	3,7(11)-Eudesmadiene	12.37	Terpenoids	1.10	<	-	-
11	Ibuprofen methyl ester	12.55	Phenylpropanoic acids	1.20	<	-	-
12	4-Propylresorcinol	13.18	Phenolics	<	-	13.90	1.75
13	3,4,5-Trimethoxyphenol	13.24	Phenolics	0.71	-	1.99	5.24
14	β-Guaiene	13.69	Terpenoids	0.78	2.22	-	-
15	Homovanillic acid	13.79	Phenolics	1.06	-	13.39	3.26
16	(−)-Spathulenol	15.30	Terpenoids	<	0.92	-	-
17	Methyl hinokiate	16.62	Terpenoids	<	2.05	-	-
18	Ylangenol	17.07	Terpenoids	<	1.00	-	-
19	Acetyltributyl citrate	19.78	Carboxylic acids	-	1.89	-	-
20	Stigmasterol	27.86	Steroids	<	2.41	-	-
21	γ-Sitosterol	28.56	Steroids	0.89	6.52	-	-
22	β-Amyrin	29.12	Terpenoids	41.45	29.51	-	-
23	Not identified	29.32	Not identified	7.41	9.49	-	-
24	α-Amyrin	29.72	Terpenoids	19.17	20.18	-	-

RT, retention time; -, not detected; <, trace of peak area that was lower than 0.5%.

## Supplementary Materials

The following are available online, Figure S1: GC-MS chromatography of crystal from fractions F15–F18.
