# Supplementary figures and images for: Antioxidant, α-Amylase and α-Glucosidase Inhibitory Activities and Potential Constituents of Canarium tramdenum Bark
Source: Molecules. 2019 Feb 9;24(3):605. doi: 10.3390/molecules24030605 (PMC6385046; doi:10.3390/molecules24030605)

## SUPPLEMENTARY DATA

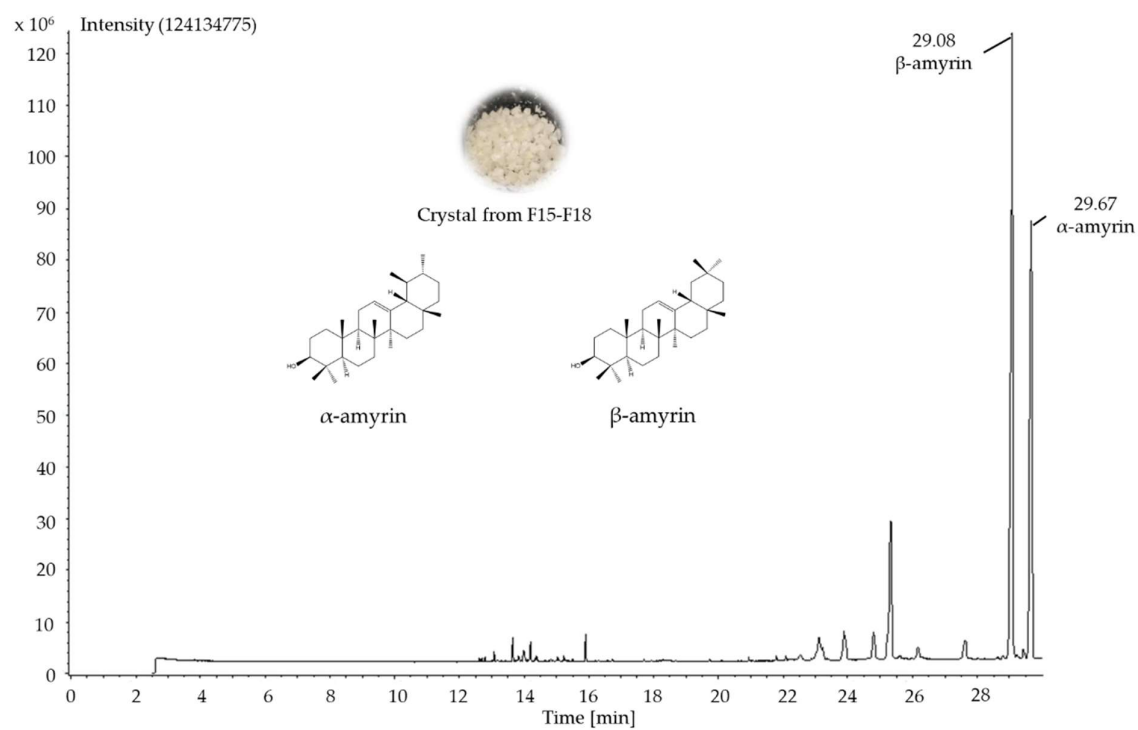

**Figure S1.** GC-MS chromatography of crystal from fractions F15-F18

Supplement: Supplementary file 1 [file molecules-24-00605-s001.pdf]
